# New Anæsthetic

**Published:** 1866-10

**Authors:** 


### New Anæsthetic.

Dr. B. F. Gilman, introduced as late Surgeon of the U. S. Army, presented to the Chicago Medical Society several bottles of a new anæsthetic, with a circular describing its properties.

He claimed for it all the advantages of chloroform, ether or nitrous oxyde, without danger of producing nausea, vomiting or headache. The agent was a compound of meconic acid, kinic acid, codeia and thein, dissolved in alcohol and formyl.

The remarks of Dr. Gilman and his circular gave rise to considerable discussion. Objection was expressed to the name of "Narcotina," which he applied to this new agent, as being a term already given to one of the active principles of opium. Objection was also made to the following phrases in the circular: Narcotina "is an almost instantaneous cure (?) for headache and neuralgie." "*It is free from all danger, and may safely be used by children and adults.*" Doubt was felt how far the following was absolutely correct: "It has been thoroughly tested in hundreds of cases, and is used in the U. S. Army, in hospitals in New York, Philadelphia and Boston." The question was asked to what extent codeia, thein and such agents would be vaporized and enter the lungs, by inhaling their solutions, poured on linen or sponge.

On motion, the subject was referred to a committee, consisting of Drs. Bogue, Bevan, Lyman, of Cook County Hospital, and Holmes, with the request that the committee should make use of this anæsthetic in such patients as they might find convenient, and report at some future meeting regarding its efficacy.

